# Dynamics of Mismatch and Alternative Excision-Dependent Repair in Replicating *Bacillus subtilis* DNA Examined Under Conditions of Neutral Selection

**DOI:** 10.3389/fmicb.2022.866089

**Published:** 2022-06-30

**Authors:** Adriana G. Patlán-Vázquez, Víctor M. Ayala-García, Carmen Vallin, Jonathan Cortés, Suria G. Vásquez-Morales, Eduardo A. Robleto, Evgeny Nudler, Mario Pedraza-Reyes

**Affiliations:** ^1^Division of Natural and Exact Sciences, Department of Biology, University of Guanajuato, Guanajuato, Mexico; ^2^Faculty of Chemical Sciences, Juarez University of Durango State, Durango, Mexico; ^3^School of Life Sciences, University of Nevada, Las Vegas, Las Vegas, NV, United States; ^4^Biological Research Center, Universidad Autónoma del Estado de Morelos, Cuernavaca, Mexico; ^5^Howard Hughes Medical Institute, New York University School of Medicine, New York, NY, United States

**Keywords:** base deamination, DNA mispairs, mismatch repair, AER repair, maximum depth sequencing

## Abstract

Spontaneous DNA deamination is a potential source of transition mutations. In *Bacillus subtilis*, EndoV, a component of the alternative excision repair pathway (AER), counteracts the mutagenicity of base deamination-induced mispairs. Here, we report that the mismatch repair (MMR) system, MutSL, prevents the harmful effects of HNO_2_, a deaminating agent of Cytosine (C), Adenine (A), and Guanine (G). Using Maximum Depth Sequencing (MDS), which measures mutagenesis under conditions of neutral selection, in *B. subtilis* strains proficient or deficient in MutSL and/or EndoV, revealed asymmetric and heterogeneous patterns of mutations in both DNA template strands. While the lagging template strand showed a higher frequency of C → T substitutions; G → A mutations, occurred more frequently in the leading template strand in different genetic backgrounds. In summary, our results unveiled a role for MutSL in preventing the deleterious effects of base deamination and uncovered differential patterns of base deamination processing by the AER and MMR systems that are influenced by the sequence context and the replicating DNA strand.

## Introduction

In all organisms, the DNA transmitted to daughter cells must be reliably duplicated and passed to the offspring. However, during DNA replication, mispairs and insertions/deletions loops introduced in both strand templates can cause genomic infidelity (Friedberg et al., 2006). Growth-associated metabolism can elicit spontaneous hydrolysis of the exocyclic amino group of cytosine, adenine, and guanine and generate uracil (U), hypoxanthine (Hx) and xanthine (X), respectively (Ponnamperuma et al., 1961; Singer and Grunberger, 1983; Friedberg et al., 2006). These analogous bases can generate the potentially mutagenic U:G, Hx:T and X:C mispairs (Friedberg et al., 2006), and cells contend with the noxious effects of these and other mispaired bases through the activity of the mismatch repair (MMR) system (Modrich and Lahue, 1996; Kunkel and Erie, 2005; Li, 2008). This repair system improves the fidelity of DNA synthesis by aborting illegitimate recombination and removing mispairs and insertion or deletions loops generated during replication (Kunkel and Erie, 2005; Li, 2008). Moreover, the MMR system ensures genome fidelity and fixes mispairs by discriminating between the old and newly synthesized strands. In *Escherichia coli*, the MMR system is composed of the proteins MutS, MutL and MutH (Kunkel and Erie, 2005; Friedberg et al., 2006; Li, 2008). This pathway is complemented by the UvrD helicase as well as a methylation system which directs the proper correction of DNA mispairs by hemi-methylating the adenine residue in the sequence d(GATC), thus tagging the template DNA strand for proper correction (Kunkel and Erie, 2005; Friedberg et al., 2006; Li, 2008).

Only *E. coli* and a few related gamma proteobacteria use a methylation system as a strand-discrimination signal during MMR (Smith et al., 2001; Friedberg et al., 2006; Lenhart et al., 2016). However, in most organisms, including *B. subtilis*, the MMR system contains MutS and MutL but lacks the endonuclease MutH and a DNA methylation system to signal the proper repair of DNA mispairs (Dreiseikelmann and Wackernagel, 1981; Smith et al., 2001; Friedberg et al., 2006; Lenhart et al., 2012, 2016). In *B. subtilis* MutS plays the same role as its counterpart in *E. coli* by acting as sensors during the mismatch recognition step (Simmons et al., 2008; Lenhart et al., 2016). In both bacteria, MutL works as a link that coordinates the formation of the MutSL complex and additional proteins required to repair DNA mispairs (Lenhart et al., 2012, 2016). However, while MutL from *B. subtilis* possesses endonuclease activity, such function is absent in the *E. coli* MutL homolog (Pillon et al., 2010; Lenhart et al., 2016). It has been proposed that single-strand breaks, produced during processing of Okazaki fragments during the DNA synthesis in the lagging strand, provide entry points for MMR components and correct repair (Friedberg et al., 2006; Lenhart et al., 2012, 2016). Nevertheless, the mechanism that generate access points for MutS/MutL in the leading strand is currently unknown. Interestingly, in *Streptococcus pneumoniae* the presence of uracil in the DNA strand that contains the incorrect base increases repair efficiency of mispairs (Mèjean et al., 1991). Furthermore, in B cells of mice and humans, the activation-induced cytidine deaminase (AID) converts cytosine to uracil, generating multiple dU:dG mispairs. Accordingly, it was proposed that single-strand breaks, formed during uracil processing, function as entry points for MMR-dependent repair (Schrader et al., 2007; Schanz et al., 2009).

Base deamination is counteracted in *B. subtilis* through, (i) the base excision repair (BER) pathway, which employs a unique DNA glycosylase to repair uracil (López-Olmos et al., 2012), (ii) an alternative, promiscuous excision repair (AER), that removes all deaminated bases and AP sites employing EndoV (Patlán et al., 2019), and (iii) Aag (a homolog of AlkA) which together with EndoV protects *B. subtilis* from the mutagenic effects of hypoxanthine (Ayala-García et al., 2016). Notably, it has been shown that EndoV plays a more prominent role than Ung in protecting *B. subtilis* from spontaneous and deamination-induced mutagenesis (López-Olmos et al., 2012; Patlán et al., 2019).

In this work, we report that the combined activities of EndoV and MutSL counteract the cytotoxic and mutagenic effects of base deamination. Furthermore, results from Maximum Depth Sequencing (MDS), which examines mutagenesis in conditions of neutral selection, revealed an increase in base deamination-promoted mutagenesis in growing *B. subtilis* cells deficient for MutSL and EndoV. Remarkably, our results suggests that C → U and G → X induced lesions were asymmetrically processed in both replicating strands. Overall, the MDS analyses showed asymmetric patterns, sequence context effects in base deamination, and their differential repair by the MutSL and EndoV-dependent systems in the lagging and leading strands of replicating *B. subtilis* cells.

## Results

### EndoV and MutSL Protect *Bacillus subtilis* From the Nitrous Acid Cytotoxicity

**Figure 1:**
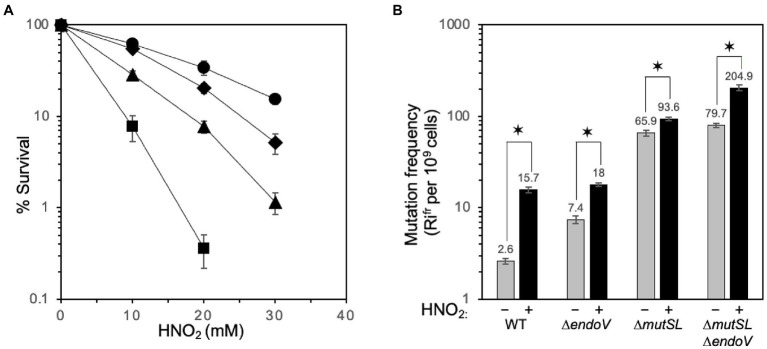
Contribution of MutSL and EndoV in protecting *Bacillus subtilis* from the cytotoxic and mutagenic effects of spontaneous and HNO_2_-promoted base deamination. **(A)** Susceptibility of different strains of *B. subtilis* to nitrous acid. *B. subtilis* WT (●), *mutSL* (♦), *endo V* (▲), and *mutSL endoV* (■) strains were grown in A3 medium to a OD_600nm_ of 0.5 and then treated with different doses of nitrous acid (HNO_2_). The results are expressed as averages ± SD of at least three independent experiments per triplicate. **(B)** Spontaneous and HNO_2_-induced mutation frequencies of strains with distinct genotypes. The strains indicated were grown at 37°C in A3 medium to an OD_600_ of 0.5 and then divided into two Erlenmeyer flasks; one of the flasks was left as an untreated control (gray bars), and the other was supplemented with an LD_50_ of HNO_2_ (black bars). The cultures were shaken for 1 h and after eliminating the deaminating agent from the amended cultures, all the flasks were shaken for an additional period of 12 h at 37°C. Finally, all the cultures were processed to calculate the frequencies of mutation to Rif^r^, as described in Materials and Methods. Each bar represents the mean of data collected from three independent experiments, each performed in sextuplicate, and the error bars represent SEMs. The asterisks indicate values that were significantly different (*, *p* < 0.05).

A previous report suggested that, in addition to Ung and EndoV, the MMR machinery of *B. subtilis* counteracts the mutagenic effects of uracil and possibly other deaminated bases (López-Olmos et al., 2012). Therefore, we investigated a possible functional relationship between the MMR system, encoded in the *mutS*-*mutL* operon (Kunst et al., 1997) and EndoV (*ywqL*) in preventing the cytotoxic effects of mispairs generated by DNA base deamination. To this end, *B. subtilis* strains proficient (WT) and deficient in MutSL, EndoV, or both repair systems were challenged with increasing doses of nitrous acid, a deaminating agent that preferentially targets adenine and cytosine over guanine (Frankel et al., 1980; Hartman et al., 1994). Results revealed that *B. subtilis* cells deficient for EndoV were more susceptible to HNO_2_ than cells of the parental wild-type strain (Figure 1A). Interestingly, disruption of *mutSL* also sensitized the cells to HNO_2_, and sensitivity of this strain increased further in the absence of *endoV* (Figure 1A). The medial lethal dose (LD_50_) of HNO_2_ for WT, Δ*endoV*, Δ*mutSL* and Δ*endoV* Δ*mutSL* strains was 18 ± 1.41, 5.6 ± 0.455, 11 ± 0.815 and 3.3 ± 0.355 mM, respectively (Supplementary Figure S1). Altogether, these results revealed that *B. subtilis* EndoV and MutSL work in independent pathways to counteract the cytotoxic effects of the DNA deaminating agent HNO_2_.

### EndoV and MutSL Counteract the Mutagenic Effects of Nitrous Acid

We tested whether EndoV and MutSL prevent the genotoxic effects of the mismatches promoted by base deaminating lesions. To this end, we determined the mutation frequency to rifampicin resistance (Rif^r^) in the presence or absence of the deaminating agent HNO_2_ in *B. subtilis* wild type and strains deficient in EndoV, MutSL, or both repair pathways. The absence of EndoV, MutSL, or both increased spontaneous Rif^r^ mutagenesis ~2.5, ~26, and ~31 fold, respectively, compared to the WT (Figure 1B). Furthermore, compared to the untreated condition, nitrous acid-induced Rif^r^ mutagenesis increased by ~4, ~2.3~1.4, and ~2.6 fold in the WT, *endoV*-, *mutSL*-, and *endoV/mutSL*-deficient cells, respectively (Figure 1B). Of note, in agreement with a previous report revealing a role for EndoV in counteracting the full spectrum of deaminated bases and AP sites (Patlán et al., 2019), we observed a higher effect in HNO_2_-induced mutagenesis in the *mutSL endoV* mutant in comparison with the *mutSL* strain. Taken together, these results demonstrated that the MMR system prevents HNO_2_-promoted mutagenesis, and that both repair pathways may additively prevent mutagenesis caused by deaminated DNA base analogs. However, additional pathways preventing base deamination, including Aag and Ung (López-Olmos et al., 2012; Ayala-García et al., 2016), could be operating in *B. subtilis*, as we observed a discrete increase in mutagenesis after disrupting *endoV* in the *mutSL*-deficient strain and following HNO_2_ treatment of the Δ*endoV* strain (Figure 1B).

### Mutation Frequencies Are Different for the Leading and Lagging Strands of EndoV and/or MutSL-Deficient Cells

**Figure 2:**
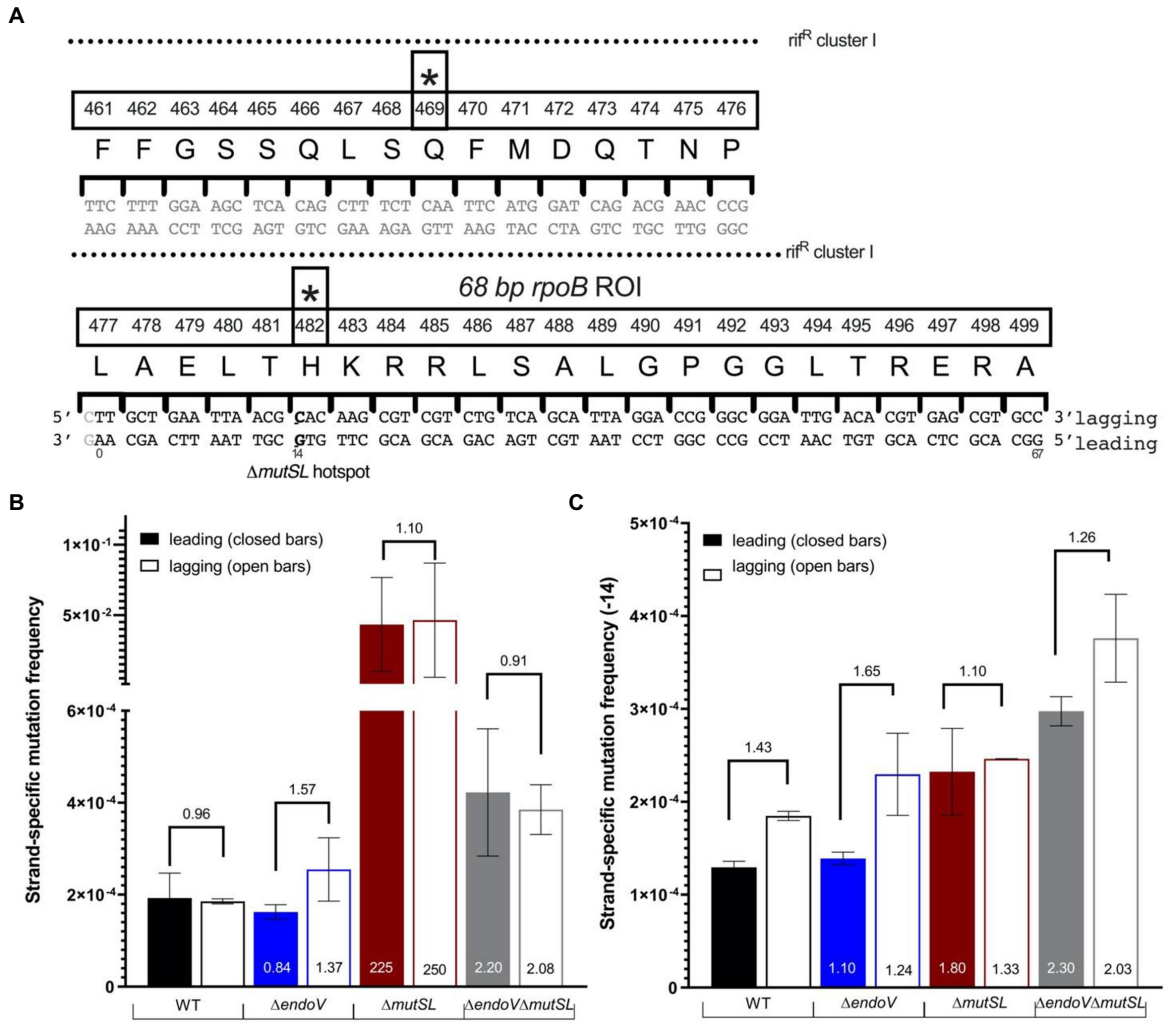
Maximum Depth Sequencing (MDS) of *B. subtilis* strains with distinct genotypes. **(A)** Schematic and sequence of the 68-bp *rpoB* ROI selected for MDS. The diagram depicts the overlap with the Rif^R^ cluster I (dotted lines), amino acid position, and the stars denote the two characterized mutagenic hotspots that confer Rif^R^ in *B. subtilis*. **(B)** Strand-specific mutation frequencies for the strains indicated. **(C)** Strand-specific mutation frequencies excluding position 14 corresponding to the first position of codon 482. The bars represent the average strand-specific frequency of two biological replicates. Each strand-specific frequency for each replicate was calculated by taking the total of all the changes in the ROI/important nodes. Error bars are SEM. Numbers on top of the graph denote the fold change increase or decrease of the lagging strand compared to the leading (example Δ*endoV* lagging/Δ*endoV* leading) while the numbers at the bottom represent the fold change increase or decrease of each strand compared to the WT (example Δ*endoV* leading/WT leading).

We investigated the strand-specific mutation frequencies in cells proficient and deficient in MMR (MutSL) and AER (EndoV) using MDS. MDS is a powerful sequencing technique that detects rare strand-specific mutations in a bacterial population under conditions of neutral selection (Jee et al., 2016). In contrast to traditional barcoding protocols (Kinde et al., 2011; Schmitt et al., 2012; Lou et al., 2013) that include an exponential amplification step to increase sequencing yield of the Region Of Interest (ROI), MDS first adds unique barcodes directly to the genomic copy of ROI and begins with a round of linear PCR, followed by exponential PCR amplification and high-throughput sequencing of the strand libraries (Supplementary Figure S2). Employing this strategy, only the ROI strand is copied initially eliminating random mutation due to polymerase errors and substantially reducing sequencing errors (Jee et al., 2016). To avoid head-on collisions between the replisome and the transcriptional machinery (Paul et al., 2013), MDS experiments were performed targeting a 68 bp sequence of the *rpoB* gene (ROI) whose transcription is co-directional with DNA replication of the *B. subtilis* genome (Figure 2A; Kunst et al., 1997). Therefore, the leading template corresponds to the transcribed strand while the lagging template corresponds to non-transcribed strand. The *rpoB* ROI overlaps with Rif^R^ cluster I and covers one of the two known mutation hotspots for *B. subtilis* growing cells (Figure 2A; Maughan et al., 2004; Perkins and Nicholson, 2008; Valenzuela-García et al., 2018). Of note, we interpret our results with and without the hotpot. These experiments were applied to genomic libraries generated from two independent cultures propagated for >90 generations of each strain. The mutation frequencies calculated from a minimum of 2 million barcode families of each independent duplicate library, gave similar values, attesting for the reproducibility of the method considering or not the hotspot (Supplementary Figure S3).

Our results revealed similar mutation frequencies in the lagging and leading template strands of the three strains analyzed (Figure 2B). The absence of a functional MMR system increased the mutation frequency by approximately ~225–250-fold compared to the parental wild-type strain in both template DNA strands (Figure 2B). Further analysis of the MDS data showed that mutagenesis in the first position of the *rpoB* codon 482 (5′**C**AC3′; Figure 2A) had a dramatic impact (hotspot) in the mutagenic levels of the *mutSL* strain; even with this datum omitted, Figure 2C indicates that MutSL deficiency produced a higher mutation frequency than the WT strain, most notably in the leading template. The disruption of *mutSL* in the *endoV*-deficient strain increased mutagenesis ~2 fold in both DNA strands compared to the wild-type strain (Figure 2C; 2.3 and 2.0-fold for the leading and lagging strands, respectively). Of note, the WT showed a slight increase in mutation frequency in the lagging strand compared to the leading strand. The same response was observed in the *endoV*^−^ and *endoV*^−^ *mutSL*^−^ strains but not in the *mutSL*^−^ strain.

These results strongly suggest that (i) MutSL and EndoV are crucial to the repair of mispairs promoted by base deamination in both DNA strands and, (ii) EndoV-dependent repair of deaminated bases can be activated in the absence of the MMR system.

### Deaminated Bases in the Leading and Lagging DNA Strands Are Differentially Processed by EndoV and MutSL

**Figure 3:**
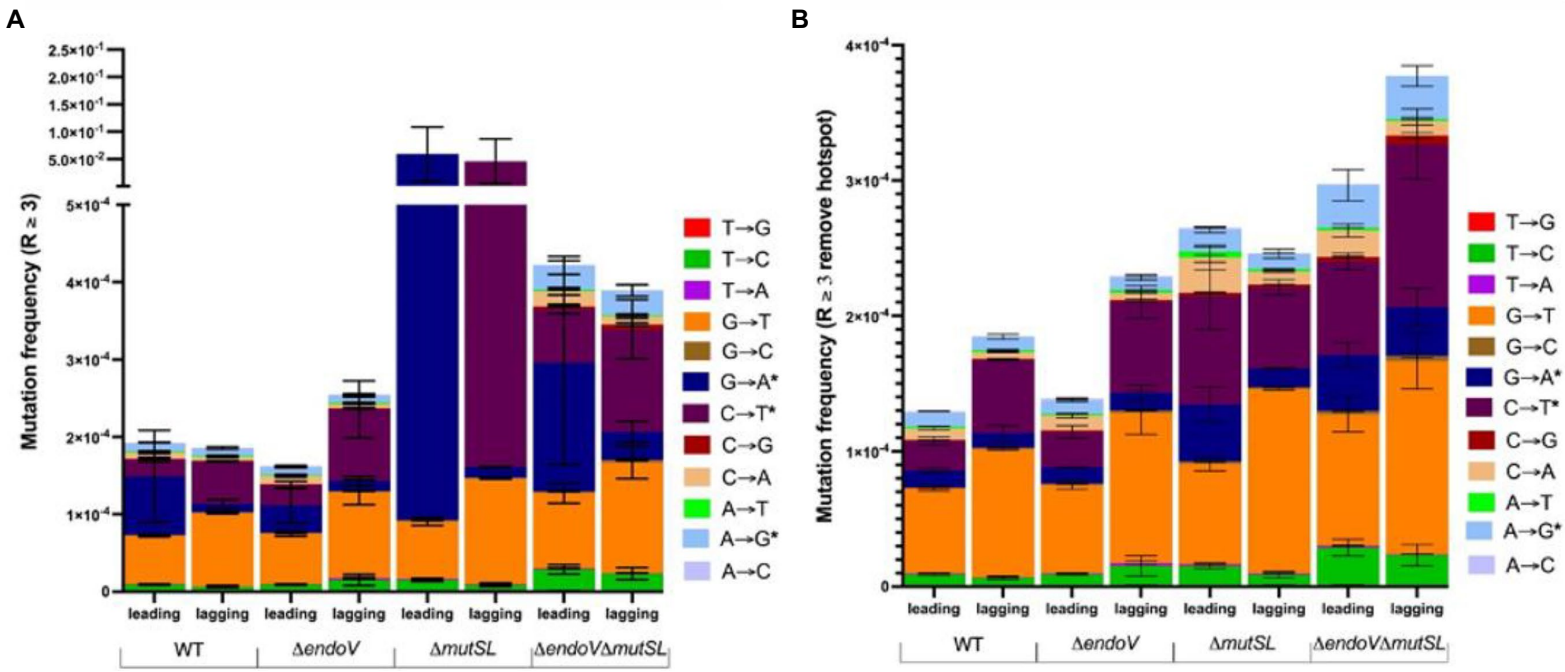
**(A)** Mutation frequencies for the strains indicated, depicting the contribution of base substitutions produced from base deamination (*) and from other types of base damages. **(B)** Mutation frequencies as shown in A but excluding position 14 corresponding to the first position of codon 482. Each section of the bars represents the mean frequency of two biological replicates. The frequency of each replicate was calculated using the sum of the specific change in the 68-bp ROI divided by the number of important/nodes or families. Error bars are SEM.

We investigated the contribution of base substitutions produced from base deamination and other base modifications to the strand-specific mutation frequency using MDS. Overall, except for the MMR-deficient strain, the results revealed that replication errors and/or base deamination-induced mutations occurred at similar levels in both DNA chains, as those elicited by other type of factors (Figure 3A). Such observations were more evident after subtracting the mutagenic levels contributed by the hotspot 482; Figure 3B shows the full spectrum of mutations detected by MDS and indicates that these events occurred asymmetrically in both DNA strands. Of note, analyses of the full spectrum of mutations detected by MDS, revealed that among the base substitutions different from those generated by base deamination, the transitions G → T predominated in both replicating DNA strands (Figure 3B; Supplementary Figure S4). Furthermore, in both biological replicates, the proportion of these transitions were maintained when we increase the number of reads per barcode (R; Supplementary Figure S4).

**Figure 4:**
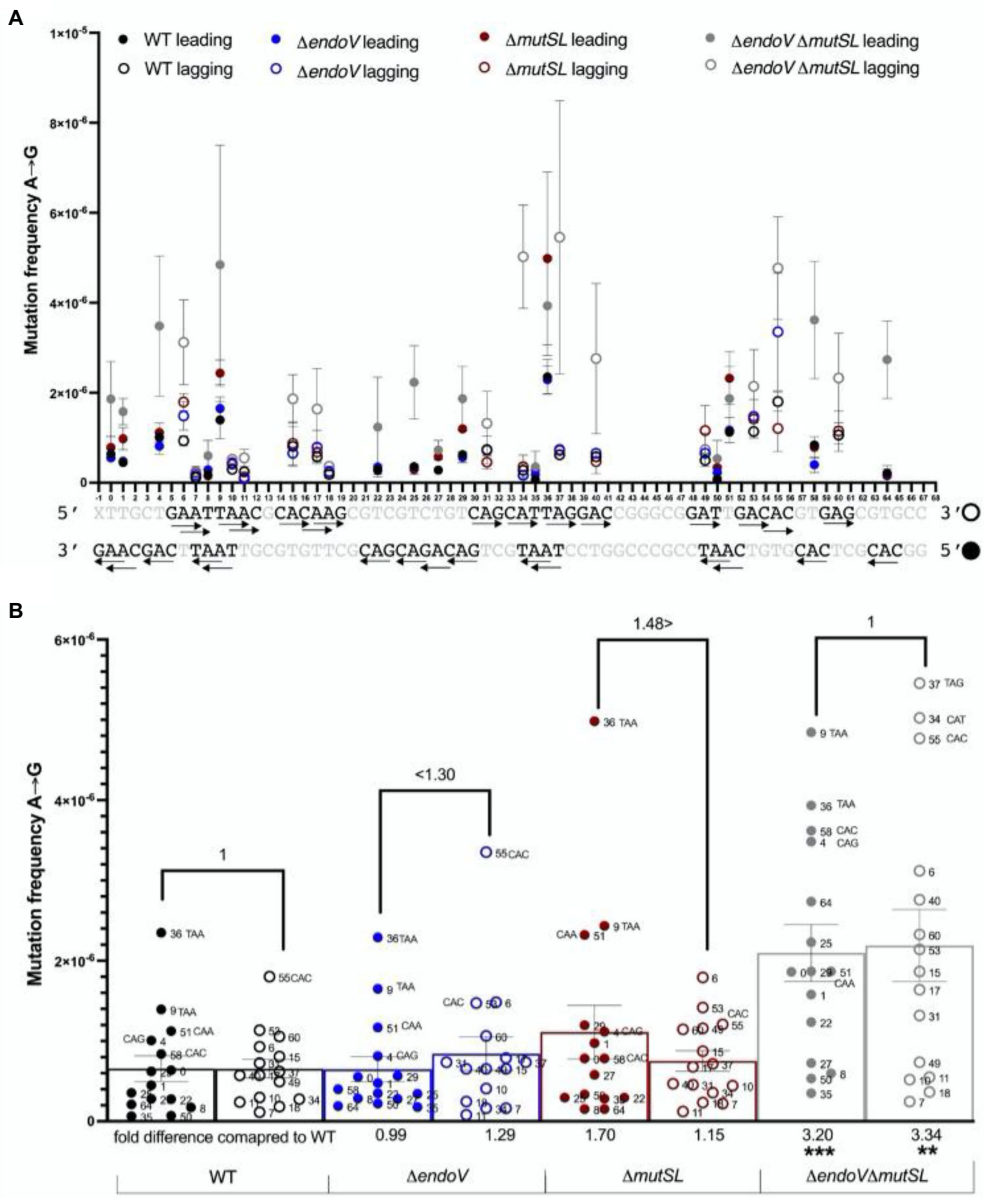
Maximum Depth Sequencing of A→G substitutions in the leading and lagging strands of the *B. subtilis* strains indicated. **(A)** A→G Mutation frequency of every A position in each strand of the 68-bp ROI. The arrow → follows 5′ → 3′ polarity. Closed circles represent the leading strand, while open circles represent the lagging strand. **(B)** The bars represent the average frequency across the ROI considering the frequency of every A position (dots). Each dot represents the average frequency of two biological replicates. Error bars are SEM. Values, and symbols >; <, above connecting lines indicate fold differences of mutation frequencies between the leading and lagging strands of each strain. Values in X axis, compare fold differences of mutation frequencies between the leading and lagging strands of the mutant strains with those from the WT. **, *p* < 0.0097; ***, *p* < 0.0002 (by the Mann–Whitney test).

We then focused our attention to the base transitions generated by each type of deaminated base at specific nucleotide positions. The WT parental strain, the *endoV*, and *mutSL* mutants displayed a similar frequency of the A → G transition in both DNA strands (Figures 4A,B). However, in comparison to the wild-type strain, the strain with combined defects in EndoV and MutSL showed an increase in this type of transition by 3-fold in both DNA strands (Figures 4A,B—see fold change in the X axis). The effects in the strains with deficiencies in EndoV or MutSL were not significantly different with respect to the WT strain (Figures 4A,B). Interestingly, the base substitution analysis also showed a high contribution of A → G transitions when A residues were at the 5′ of pyrimidines (Figure 4A). Accordingly, the triplets, TAA > CAC > CAG in the leading and, TAG > CAT > CAC, in the lagging strands, showed the highest frequencies of adenine to guanine substitutions in strains with deficiencies in MutSL and/or EndoV (Supplementary Figure S5).

**Figure 5:**
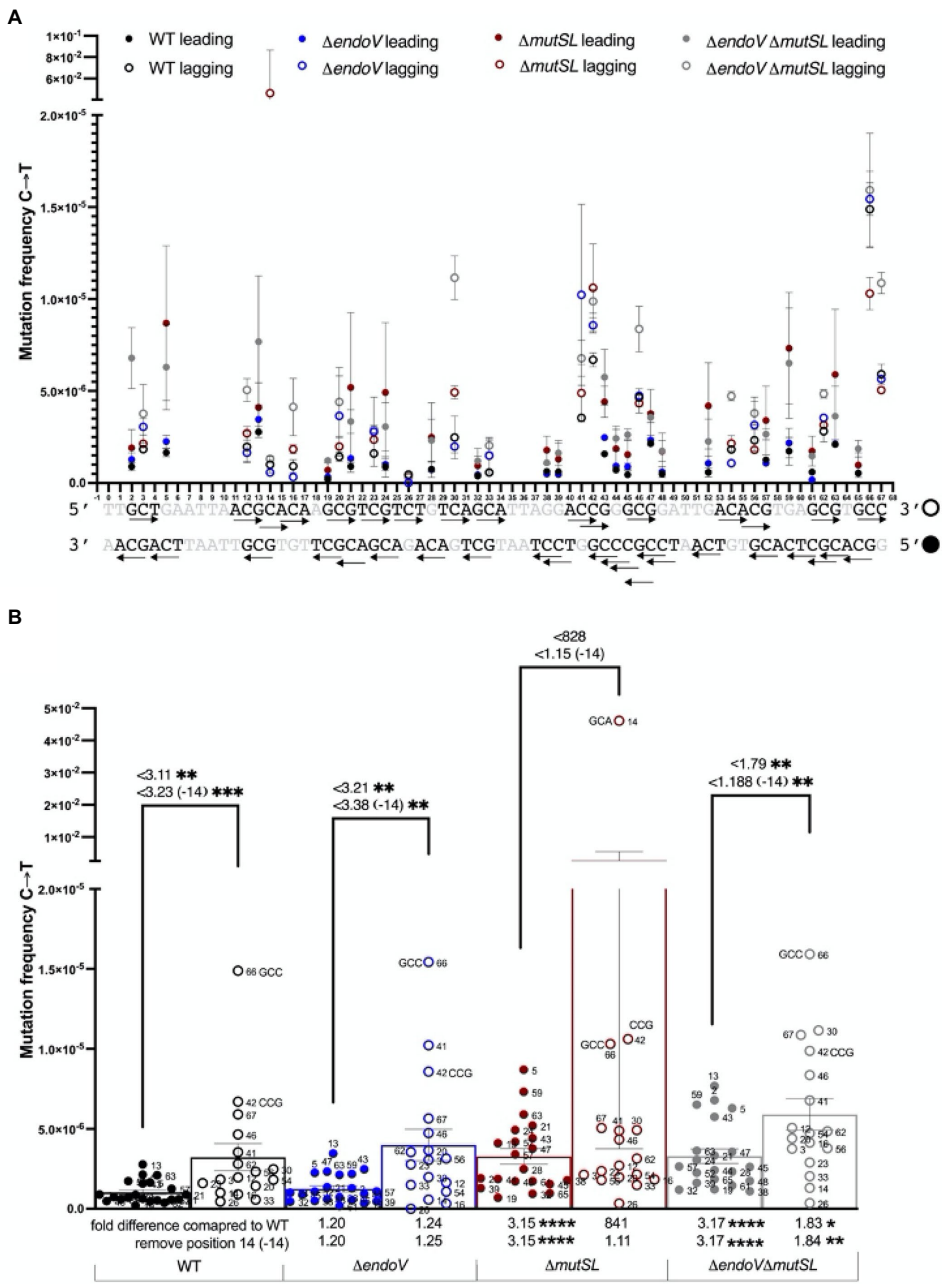
Maximum Depth Sequencing of C→T substitutions in the leading and lagging strands of the *B. subtilis* strains indicated. **(A)** C→T Mutation frequency of every A position in each strand of the 68-bp ROI. The arrow → follows 5′ → 3′ polarity. Closed circles represent the leading strand, while open circles represent the lagging strand. **(B)** The bars represent the average frequency across the ROI considering the frequency of every C position (dots). Each dot represents the average frequency of two biological replicates. Error bars are SEM. Values (considering or not the contribution of the hot-spot 14), and symbol <, above connecting lines indicate times differences of mutation frequencies between the lagging and leading strand of each strain. Values in X axis, compare times differences of mutation frequencies (considering or not the contribution of the hot-spot 14) between the leading and lagging strands of the mutant strains with those from the WT. *, *p* < 0.01; **, *p* < 0.0097; ***, *p* < 0.001; ****, *p* < 0.0001 (by the Mann–Whitney test).

Our MDS analyses revealed that except for the MMR-deficient strain, a significant increase of the C → T transitions in the lagging strand compared to C → T transitions in the leading strand was observed in the other strains analyzed (Figures 5A,B). Of note, the frequency of this transition increased further in the Δ*mutSL* strain ~828 times and such effect was contributed by the first cytosine located in the codon 482 (5′**C**AC3′), which is reported to be a hotspot in *B. subtilis* (Valenzuela-García et al., 2018). Our subsequent analysis with this position eliminated showed that the lagging strand of the WT and *endoV* strains still contained a significant higher amount of C → T transitions than the leading strand (~3 fold), while the effect diminished in the *endoV mutSL* strain (~1.188; Figures 5A,B). As shown in Figure 5B; Supplementary Figure S6, this effect was dependent on the sequence context as the GCA, GCC and CCG triplets were mostly involved in conferring this type of transition.

In reference to the WT, the strain with combined MMR and AER deficiencies exhibited higher mutation frequencies of C → Ts in both DNA strands (Figure 5B—see fold change in the X axis). The frequency of this transition also increased significantly, but only in the leading strand of the *mutSL* strain (Figure 5B). Of note, similar results were observed after eliminating the contribution of the hot spot C → T in the position 14 of the ROI analyzed (Figure 5B).

**Figure 6:**
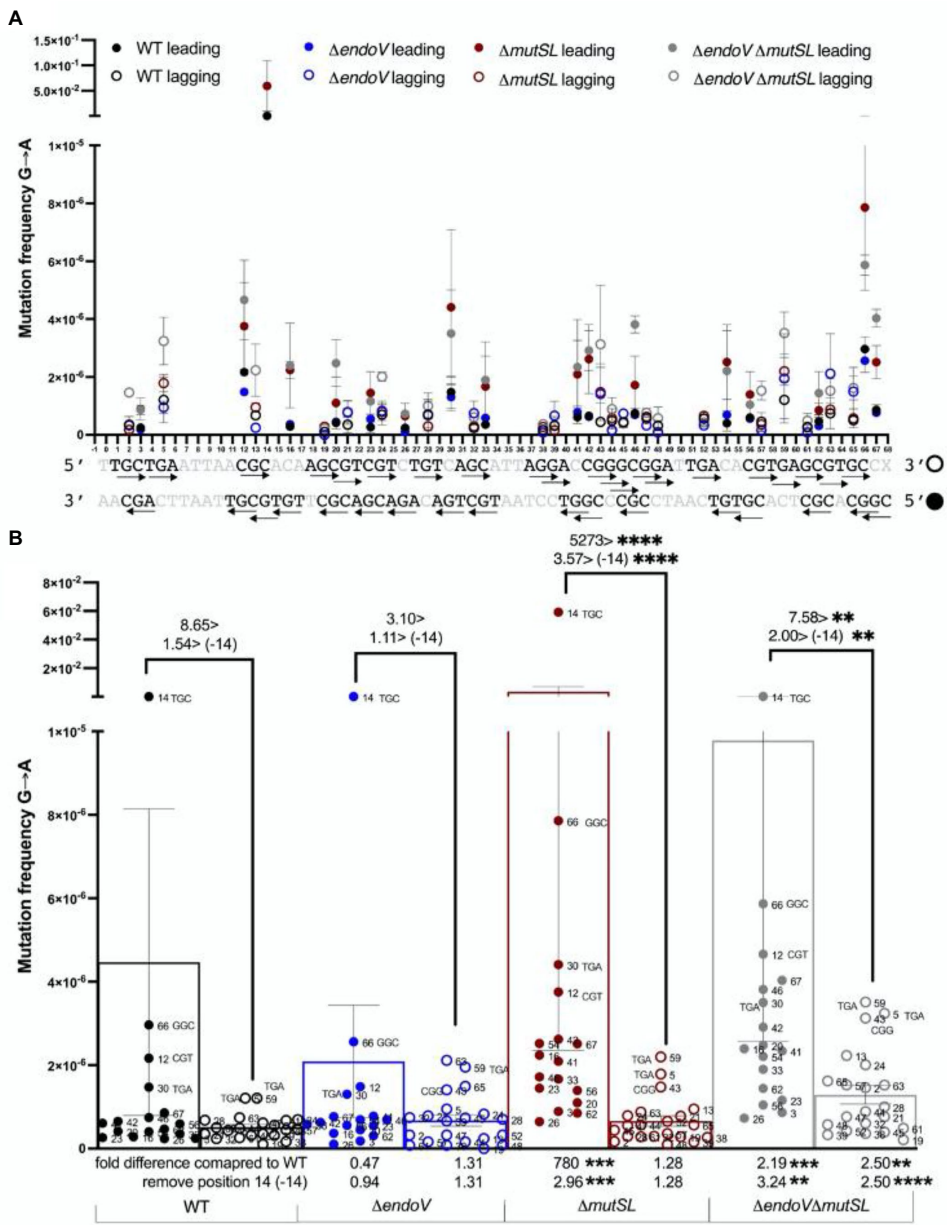
Maximum Depth Sequencing of G→A substitutions in the leading and lagging strands of the *B. subtilis* strains indicated. **(A)** G→A Mutation frequency of every G position in each strand of the 68-bp ROI. The arrow → follows 5′ → 3′ polarity. Closed circles represent the leading strand, while open circles represent the lagging strand. **(B)** The bars represent the average frequency across the ROI considering the frequency of every G position (dots). Each dot represents the average frequency of two biological replicates. Error bars are SEM. Values (considering or not the contribution of the hot-spot 14), and symbol >, above connecting lines indicate times differences of mutation frequencies between the leading and lagging strand of each strain. Values in X axis, compare times differences of mutation frequencies (considering or not the contribution of the hot-spot 14) between the leading and lagging strands of the mutant strains with those from the WT. **, *p* < 0.0014 (above) and *p* < 0.0014 (below); ***, *p* < 0.0002; ****, *p* < 0.0001 (by the Mann–Whitney test).

Further MDS results showed that the *mutSL* and *mutSL endoV* strains produced a significant higher level of G → A substitutions in the leading strand, a change that can occur by replication errors and guanine deamination to xanthine resulting in the generation of X:T mispairs (Figures 6A,B). Also, we found that in the MutSL-deficient strain, the third base in the codon 482 (3′**G**TG5′) represented a hot spot of the G → A mutations (Figures 6A,B); notably, these transitions were EndoV-dependent as the contribution of this hot spot to mutagenesis significantly decreased in the MMR/AER-deficient strain (Figures 6A,B). It was found that after subtracting the contribution of this spot, the leading strand of the *mutSL* strain and *endoV mutSL* strain still exhibited a higher amount of G → A substitutions than the lagging strand (Figures 6A,B). Importantly, the G to A mutations occurred more frequently at TGC and CGG sequences than in other G-containing triplets in the leading strand, while in the lagging strand such substitutions were more frequent at TGA and GGC triplets (Supplementary Figure S7). Of note, with respect to the WT, the frequencies of the transition G → A were significantly higher in the leading strand of the *mutSL* strain and in both DNA strands of the MMR/AER-deficient strain (Figure 6B—see fold change in the X axis).

Overall, the MDS analyses showed asymmetric patterns, sequence context effects in C → T and G → A mutations, and their differential repair by the MutSL and EndoV-dependent systems in the lagging and leading strands of replicating *B. subtilis* cells. These contentions are further supported by experiments that compared frequencies of C → T substitutions directly to its complementary G → A substitution in both DNA strands, as shown in Supplementary Figures S8A**–**D.

## Discussion

In this work, we showed that MMR and EndoV confer protection to *B. subtilis* from the cytotoxic and/or mutagenic effects of a deaminating agent of DNA. Furthermore, MDS analysis of C→T, G→A and A→G substitutions that can result from base deamination-induced mispairs or replication errors revealed asymmetric and sequence context patterns of mismatch and alternative excision-dependent repair in replicating DNA strands of *B. subtilis* cells.

In *B. subtilis* the MMR repair pathway contributes to the prevention of mutations caused by dU:dG mispairs (Frankel et al., 1980); interestingly, a similar role has been attributed to this pathway in mammals (Schrader et al., 2007; Schanz et al., 2009). Here, we report that MMR in *B. subtilis* processes a broader range of DNA mispairs. Accordingly, C → T, G → A and A → G substitutions were detected by MDS, and analysis of mutation frequencies, suggested an U:G > X:T > Hx:C, hierarchical order of MMR recognition and processing (Figures 1, 3). While G→A, A→G and C→T transitions can result from replication errors that escape MMR (Ayala-García et al., 2016), these mutations occur spontaneously or through enzymatic deamination events (Wang et al., 1998; Krokan et al., 2002; Friedberg et al., 2006; Chatterjee and Walker, 2017). Accordingly, a significant proportion of the C→T and the hotspot G→A and C→T mutations that arose in the MMR-deficient strain were dependent on EndoV (Figures 4, 6).

While MDS has been successfully applied to determine mutation rates in bacteria (Jee et al., 2016), its versatility has been recently proven in the elegant work of Li et al. (2020) who employed this technique to identify the urethane-inducing dominant Kras Gln_61_-Ile mutation in mouse lungs. In this report, to better understand the dynamics of base deamination repair, at DNA strand level, we applied MDS analysis to cultures of strains proficient or deficient for MutSL, EndoV, and both repair systems. Notably, under conditions of neutral selection, MDS detected the C→T transition in the codon 482 (5′**C**AC3′) of the *rpoB* ROI employed. This mutation was previously identified in *B. subtilis* as a hotspot in colonies that acquired a phenotype of rifampin resistance (Campbell et al., 2001; Valenzuela-García et al., 2018). While this substitution has been detected in vegetative cells and outgrowing spores of *B. subtilis* (Maughan et al., 2004; Valenzuela-García et al., 2018), no selective advantage has been associated to this mutation. Accordingly, the growth rate, spore germination or metabolic properties of *B. subtilis* cells carrying the predicted aminoacidic change H_482_Y were not severely affected in comparison with cells or spores of the wild-type parental strain (Maughan et al., 2004; Perkins and Nicholson, 2008).

It has been shown that in addition to EndoV and MutSL, Ung counteracts the mutagenic effects of cytosine deamination in *B. subtilis* (López-Olmos et al., 2012). In agreement with this observation, our MDS analyses revealed that the simultaneous disruption of *endoV* and *mutSL* was necessary to increase the frequency of C → T transitions in both DNA strands (Figure 3B). Furthermore, we found that while strains exhibited similar mutation frequencies in both DNA strands, further analyses indicated that repair of specific deamination-induced mispairs was strand-dependent.

Asymmetric patterns of base substitutions modulated by the genetic context have been previously reported in *B. subtilis* and other organisms (Sung et al., 2015; Long et al., 2018; Castañeda-García et al., 2020). However, our MDS experiments, which measured mutations in the leading and lagging template strands separately revealed that spontaneous C→T substitutions occurred more frequently in the lagging strand of the parental and strains deficient for EndoV and EndoV/MutSL; in contrast, G→A mutations promoted by guanine deamination happened more frequently in the leading than in the lagging strand of the *mutSL* and *endoV mutSL* strains. However, it must be pointed that MDS has limitations to differentiate between transient changes and true/fixed mutations in the ROI analyzed. Thus, it is possible that the C→T and G→A substitutions detected in the *rpoB* ROI represent “transient” deamination events as cells used to prepare the libraries are in continuous replication. Under this condition, base damage, including deamination and misincorporated nucleotides by the replicative machinery leading to mispairs are dynamically scanned and potentially subjected to repair during DNA replication (Supplementary Figures S8E F). The bias toward C→T substitutions in the lagging strand in *B. subtilis* is unprecedented, but intra-strand asymmetric patterns of G→A and A→G transitions were described previously in *Mycobacterium smegmatis* and *E. coli* (Lee et al., 2012; Castañeda-García et al., 2020). Taken together, our results support the notion that cytosine and guanine deamination take place asymmetrically in both DNA chains and unveiled differential patterns of mismatch and alternative excision-dependent repair of base-deaminated promoted mispairs.

Other deep-genome sequencing experiments examining mutation-accumulation lines in different microorganisms showed that mismatch repair of DNA transitions and transversions are affected by the neighboring base composition (Ayala-García et al., 2016; Castañeda-García et al., 2020). Employing MDS, here we found that the sequence context and DNA strand influenced the levels of mutations promoted by base deamination in *B. subtilis*. Spontaneous mutations occurred more frequently at DNA sequences bearing two adjacent As, Cs or Gs. Hence, A→G substitutions occurred more frequently at TAA and CAC sequences and increased in MutSL and EndoV-deficient strains, suggesting that an adjacent pyrimidine determines the repair efficiency of deaminated adenines. Interestingly, MutSL and EndoV counteracted the A→G substitutions in both replicating DNA strands in a symmetric manner (Figures 3A,B). Strikingly, a recent report revealed that adenine deamination events in conserved promoter DNA bases elicit mutagenic events driven by head-on collisions of the transcription/replication machineries (Sankar et al., 2016). Of note, here MDS revealed that the C→T substitution happened more frequently in GCA, GCC, and CCG trimers and increased in strains deficient for MutSL and EndoV; however, such events occurred with more frequency in the lagging than in the leading strand. In agreement with these results, a significantly higher rate of C:G to T:A transitions occurred in the lagging strand of *E. coli* cells expressing the deaminase domain of the mammal APOBEC3G1 protein grown for thousands of generations (Bhagwat et al., 2016). For G→A transitions, an opposite effect was observed, as these substitutions occurred more frequently at TGC and GGC sequences located in the leading than in the lagging strand. This pattern was increased in strains with disabled MMR and AER pathways. Notably, despite the presence of 2C and 3C repeats in the leading strand as well as sequential 2G and 3G bases in the lagging chain, C→T and G→A mutations and their repair by MMR and AER pathways displayed an asymmetric behavior (Figures 5, 6). Furthermore, as noted above, a proportion of the transitions promoted by adenine, guanine and cytosine deamination generated in the MMR-deficient strain were elicited by EndoV and such events were also determined by the sequence context.

**Figure 7:**
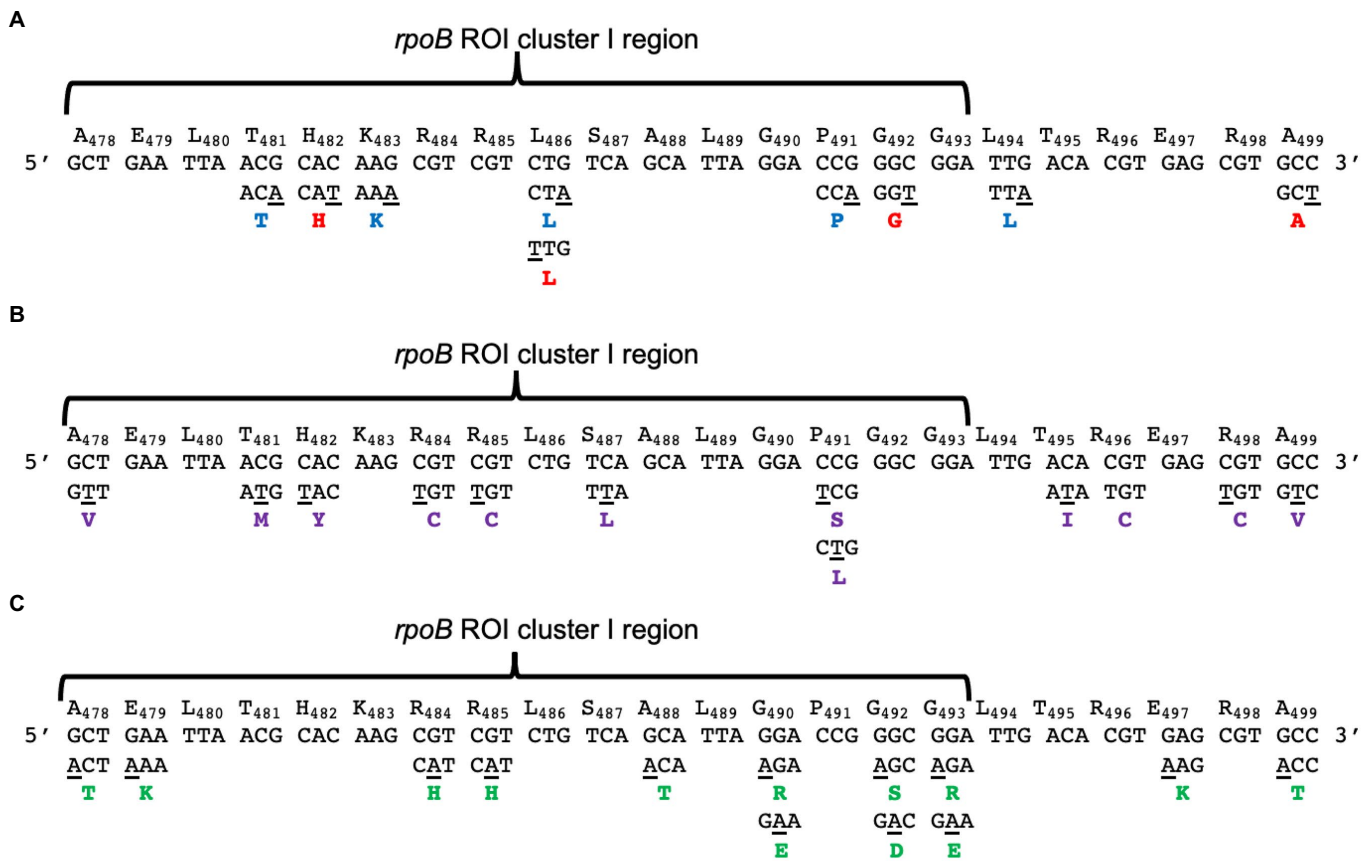
Spectrum of C→T and A→G transitions leading to silent **(A)** and aminoacidic changes **(B,C)** identified by MDS in the *rpoB* ROI. Each scheme **(A–C)** shows the WT codons and position of the encoded amino acids in the *rpoB* ROI analyzed. The base substitutions are shown underlined in the mutated codon below the WT codons. The amino acid changes (in the single letter format) are shown in bold type, below the mutated codons. Silent mutations **(A)** derived from G→A and C→T substitutions are shown in blue and red bold type, respectively. Aminoacidic changes derived from C→T mutations **(B)** are shown in purple bold type letter below each mutated codon. Aminoacidic changes derived from G→A transitions **(C)** are shown in green bold type letter below each mutated codon.

**Figure 8:**
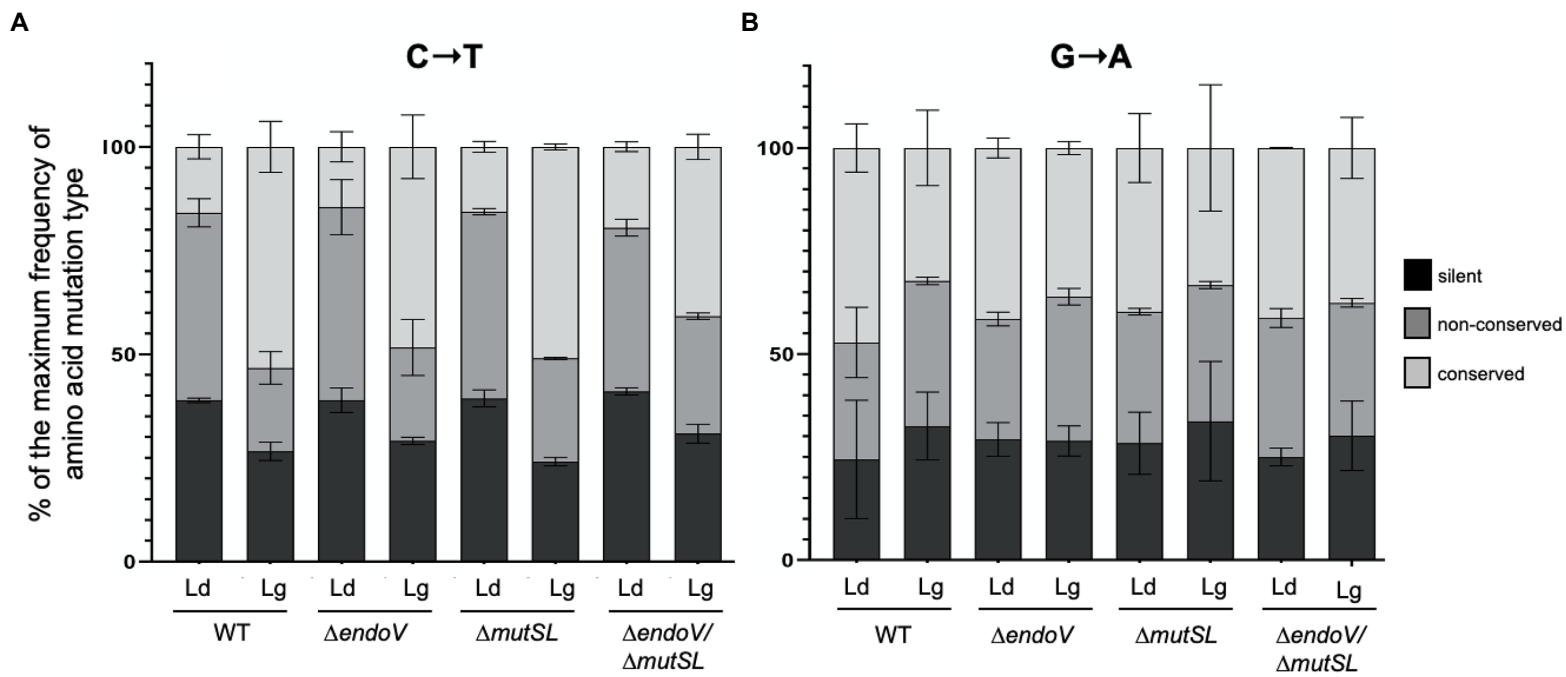
Frequency of predicted silent (black bars), non-conserved (dark grey bars) and conserved (light grey bars) amino acid changes promoted by C→T **(A)** and A→G **(B)** transitions in the leading (Ld) and lagging (Lg) strands of the *rpoB* ROI analyzed. The bars represent the average frequency of each type of silent and amino acid mutations in the leading and lagging strands across the *rpoB* ROI considering the frequency of every C→T and A→G mutation. The frequency values, in the four strains, disregard the contribution of the hot spot 14 (H_482_) in the *rpoB* ROI. Values represent the average frequency of two biological replicates. Error bars are SEM.

The asymmetry of C→T and G→A substitutions, observed by MDS, in the replicating DNA strands of the strains analyzed, prompted us to determine its impact in the predicted amino acid sequence of the *rpoB* ROI. A wide diversity of non-conserved and conserved amino acid changes derived from these substitutions were detected in the cluster I region of the *rpoB* ROI, including the previously reported hot spot H_482_→Y mutation (Campbell et al., 2001; Maughan et al., 2004; Valenzuela-García et al., 2018; Figure 7). However, novel amino acid changes were also observed in this cluster, including, R_484_→C, H; A_488_→T; G_490_→R, E; P_491_→S, L; G_492_→S, D and G_493_→R, E (Figure 7). Furthermore, our mutagenesis analyses performed under conditions of neutral selection allowed us to identify a set of silent mutations in the codons T_481_, H_482_, K_483,_ L_485_, P_490_, G_491_, G_492_, L_493_, T_494_, R_495_, E_496_, R_497_ and A_498_ (Figure 7). Additional predicted amino acid changes included, A_478_→V, T; E_479_→K; T_495_→I; R_496_→C; E_497_→K; R_498_→C and A_499_→V, T (Figure 7). Notably, disregarding the prominent contribution of the hotspot 14 [CAC; (H_482_)] in the *rpoB* ROI (Figure 2) and assuming that the transient mutations detected in the leading and lagging libraries were fixed, it was found that the C→T transversions gave rise to a similar proportion of silent, conserved, and non-conserved amino acid changes, predominating the first two, in the leading strand of all the strains analyzed; In contrast, conserved amino acid changes resulting from C→T mutations predominated in the lagging strands of strains proficient or deficient for MutSL and/or EndoV (Figure 8). Of note, G→A substitutions promoted similar proportions of silent, non-conserved and conserved amino acid mutations in both DNA strands in the wild type and mutants analyzed (Figure 8). Overall, these results emphasize the advantage of MDS versus traditional loss-of or gain-of function mutagenesis protocols in identifying the full repertoire of mutations and its frequency in replicating DNA strands in any selected ROI.

In summary, the results from this report support the following concepts, (i) spontaneous or directed mechanisms generate asymmetric patterns of C→T and G→A substitutions in the leading and lagging strands, respectively, during replication, and (ii) the sequence context influences the activity of the mismatch and alternative excision repair pathways operating over mispairs promoted by these deaminated bases.

## Materials and Methods

### Bacterial Strains and Growth Conditions

All *B. subtilis* strains used in this work were derived from strain 168 and are listed in Supplementary Table S1. The grow medium used routinely lysogeny broth (LB; Lennox formulation) or in Penassay broth (PAB; Antibiotic Medium 3; Difco Laboratories, Sparks, MD; Gerhardt, 1996). When required, the cultures were supplemented with neomycin (Neo; 10 μg ml^−1^), erythromycin (Em; 5 μg ml^−1^), chloramphenicol (Cm; 5 μg ml^−1^), spectinomycin (Spc; 100 μg ml^−1^) rifampicin (Rif; 10 μg ml^−1^) or isopropyl-β-D-thiogalactopyranoside (IPTG; 0.25 mM). Liquid cultures were incubated at 37°C with vigorous aeration (shaking at 250 rpm). Cultures on solid media were grown at 37°C. The optical density at 600 nm (OD_600_) of liquid cultures was monitored with a Pharmacia Ultrospec 2000 spectrophotometer.

### Determination of HNO_2_ Susceptibility

To determine the dose–response curves for survival of *B. subtilis* cells following HNO_2_ exposure, WT and mutant strains deficient for DNA repair systems (Supplementary Table S1) were grown at 37°C in PAB medium to an OD_600_ of 0.5. Cells were collected by centrifugation (4,800 × *g*; 10 min/25°C), washed once with phosphate buffered saline (PBS) buffer 1X (NaCl, 8 g/L; KCl, 0.2 g/L; Na_2_HPO_4_, 1.44 g/L; KH_2_PO_4_, 0.2 g/L) and adjust to an OD_600_ of 1. At the same time the nitrous acid (HNO_2_) solution was prepared mixing equimolar quantities of sodium acetate (CH_3_COONa) 4 M, pH 4.3 and sodium nitrite (NaNO_2_; 99.97% purity; JT Baker, Phillipsburg, NJ, United States) at 4 M in 500 μl of PBS 1X buffer (Tennen et al., 2000). Cell samples (500 μl) were left untreated or treated with different concentrations of HNO_2_ during 1 h at room temperature. Cell survival was measured by plating aliquots of serial dilutions on LB gar plates. CFU were counted after 16 h of incubation at 37°C.

### Determination of Spontaneous and HNO_2_-Induced Mutation Frequencies

Mutations to Rif^r^ in *B. subtilis* cells treated with nitrous acid that promote the deamination of DNA bases (Frankel et al., 1980; Hartman et al., 1994) were determined as follows. A3 cultures of each strain grown for 8 h were inoculated into flasks containing fresh A3 medium to an OD_600_ of 0.5, at this point, each culture was divided in half, and the two halves were transferred into different flasks. One of the cultures was left untreated, and the other was amended for 1 h with a lethal dose 50 (LD_50_) of HNO_2_, determined for each strain. At the end of this period, the medium was removed and replaced with fresh medium, finally, both flasks were shaken at 37°C for 12 h. Mutation frequencies were determined by plating aliquots of each culture onto six LB plates containing 10 μg/ml rifampin as well as plating aliquots of appropriate dilutions onto LB plates without rifampin. Rif^r^ colonies counted after 24 h of incubation at 37°C.

### Growth for Maximum Depth Sequencing

To determine the rare mutations that occur during replication a MDS protocol (Jee et al., 2016) was implemented in *B. subtilis*. To this end, *B. subtilis* strains WT, Δ*endoV*, Δ*mutSL* and Δ*endoV mutSL* were streaked onto LB agar from freezer stocks and grown at 30°C for 24 h. According to plating colony-forming unit (c.f.u.) counting, the average number of cells by colony is 3 × 10^8^ (thus the number of generations is ln(3 × 10^8^) = 19.5). For each strain a single colony was used to inoculate 1 ml of LB in a tube. The culture was grown at 37°C in a shaker for 6 h, it is assumed that after the transition to growing in liquid, growth occurs for ~6 generations. Four microliter (~10^7^ bacteria) were transferred to 100 ml of fresh liquid LB medium. The cultures were shaken for 24 h at 37°C, according to cell counts (for a total average of 2.35 × 10^10^ bacteria). This process was repeated 9 times. The average number of generations a bacteria would have grown in each liquid culture is


$$\ln\left(\frac{2.35x10^{10}}{10^{7}}\right)=7.76\;generations$$

Thus, the average total number of generations *g* is 19.5 + 6 + (9 × 7.76) = 95.

### Maximum Depth Sequencing

MDS was performed as previously described (Jee et al., 2016). In brief, samples of 10 ml from the last cultures of the four *B. subtilis* strains were spun down and resuspended in 1 ml Tris-EDTA buffer (pH7.5) and incubated −80°C overnight. Genomic DNA extraction was performed using Qiagen genomic tip (100G) and quantified using Nanodrop. To generate each library, 2 μg of genomic DNA were independently treated with *Nla*III (leading strand) or *Hpy*166III (lagging strand) restriction enzyme, which cleaves and delimits the Region Of Interest (ROI). The primers design was performed considering the cut sequence of *Nla*III or *Hpy*166III for leading or lagging strand, respectively. A single PCR cycle was performed with 500 ng of restriction enzyme treated genomic DNA, 500 μM barcoded forward adapter primers annealing to the 3′ end of the ROI and Q5 Hot Start polymerase (New England Biolabs; Ipswich, MA, United States). Unused barcodes were removed with *Exo*I. The forward library was amplified using a forward adapter (described below in “Primer schema”) and performing 14 cycles of PCR with Q5 Hot Start high-fidelity DNA polymerase. Reverse adapter primers were used to define the ROI. Finally reverse adapters were used to amplify the libraries in 15 cycles of PCR. All libraries were resolved in an 8% acrylamide gel and purified with Ampure XP beads (see Supplementary Figures S2, S9).

### Primer Schema

In the following sequences,

[P5] = AATGATACGGCGACCACCGA
[Rd1Seq] = GTCTACACTCTTTCCCTCACGACGCTCTTCCGATCT
[Pad] = variable length sequence (see below)
[barcode] = NNNNNNNNNNNNNN
[P7] = CAAGCAGAAGACGGCATACGA
[Rd2Seq] = GATCGGTCTCGGCATTCCTGCTGAACCGCTCTTCCGATCT
Adapter-barcode-primer: [P5][Rd1Seq][Pad][barcode][primer]
Where
*rpoB* leading [primer] = ATTGTCGTGGTTTGTATCG
*rpoB* lagging [primer] = ACCTTAGACCACTCGACGTAG
[Pad1]= g
[Pad2]= cg
[Pad3]= acg
Forward adapter amplifier:AATGATACGGCGACCACC
Adapter-reverse-primer: [P7][Rd2Seq][primer]
Where
*rpoB* leading [primer] = ACCTTAGACCACTCGACGTAG
*rpoB* lagging [primer] = ATTGTCGTGGTTTGTATCG
Reverse adapter amplifier: CAAGCAGAAGACGGCATAC

All libraries were paired-end sequenced at the NYU Langone’s Genome Technology Center on the Illumina Novaseq 6000 platform with a 20% PhiX spike in.

### Determination of Mutation Frequencies

Mutations were called as previously described (Jee et al., 2016) using an in-house code. In brief, MDS program groups reads (R) based on unique barcode to create families or important nodes. This means 1 important node = 1 barcode/1 original molecule of DNA. Families with R < 3 are filtered out and mutations are called on the rest of the families if >70% of reads have the same change at an ROI position in the same family (For a deeper explanation, please see Supplementary Figure S2). The mutation frequency of each type of transition (i.e., C→T; G→A and A→G) was determined from the total amount of each type of base change at a specific position divided by the number of important nodes and by the total number of adenines, cytosines or guanines in the ROI. The mutation frequencies shown in Figure 2 were calculated by taking the average of the strand-specific frequency of two biological replicates. Each strand-specific frequency for each replicate was calculated by taking the total of all the base changes in the ROI/important nodes. The mutation frequencies shown in Figure 3 were calculated by taking the average of the strand-specific frequency of two biological replicates for each type of mutation. Each section of the bars represents the mean frequency of two biological replicates. The frequency of each replicate was calculated using the sum of the specific change in the 68-bp ROI divided by the number of important/nodes or families. These procedures were performed independently for each library and the mutation frequencies were reported as averages of two biological replicates ± SEM. Supplementary Figure S9 shows additional information explaining MDS data interpretation for the leading and lagging strand mutation frequencies.

### Statistical Analyses

Differences in mutagenesis levels between strains and cultures treated with HNO_2_ or left untreated (Figures 1A,B; Supplementary Figure S1) were calculated by Mann–Whitney U test, and analyses were done using Minitab 17 software. Value of *p* < 0.05 were considered significant.

A comparison between the frequencies of base transitions of each mutant strain in reference to the wild type and between DNA strands was performed. To establish significant differences in these analyses, a nonparametric Mann–Whitney test, followed by Wilcoxon test (paired nonparametric *t*-test), and confirmed by additional Friedman test with a *post hoc* Dunn’s multiple comparison analysis, were performed using GraphPad Prism 8 software. Value of *p* considered significant are indicated in Figures 4–6.

## Data Availability Statement

The sequencing data will be available with the link provided. Expert personnel from Nudle’s Labr has requested to NCBI SRA Submissions Staff to fix the accession problems.

## Author Contributions

MP-R, AP-V, CV, ER, and EN designed the study and wrote the paper. AP-V, CV, VA-G, JC, and SV-M performed the research and analyzed the data. All authors contributed to the article and approved the submitted version.

## Funding

This work was supported by the National Council of Science and Technology (CONACYT; grants 221231 and A-1S-27116) of México and the University of Guanajuato (grants CIIC 082/2021 and 107/2022), the NIH grant (GM131410), and the Blavatnik Family Foundation and the Howard Hughes Medical Institute (E.N.). AP-V was supported by a scholarship from CONACyT.

## Conflict of Interest

The authors declare that the research was conducted in the absence of any commercial or financial relationships that could be construed as a potential conflict of interest.

## Publisher’s Note

All claims expressed in this article are solely those of the authors and do not necessarily represent those of their affiliated organizations, or those of the publisher, the editors and the reviewers. Any product that may be evaluated in this article, or claim that may be made by its manufacturer, is not guaranteed or endorsed by the publisher.
