# Supplementary material for: Dynamics of Mismatch and Alternative Excision-Dependent Repair in Replicating Bacillus subtilis DNA Examined Under Conditions of Neutral Selection
Source: Front Microbiol. 2022 Jun 30;13:866089. doi: 10.3389/fmicb.2022.866089 (PMC9280176; doi:10.3389/fmicb.2022.866089)
Supplement: Supplementary file 1 [file Data_Sheet_1.pdf]

Supplementary Material

Supplementary Figures

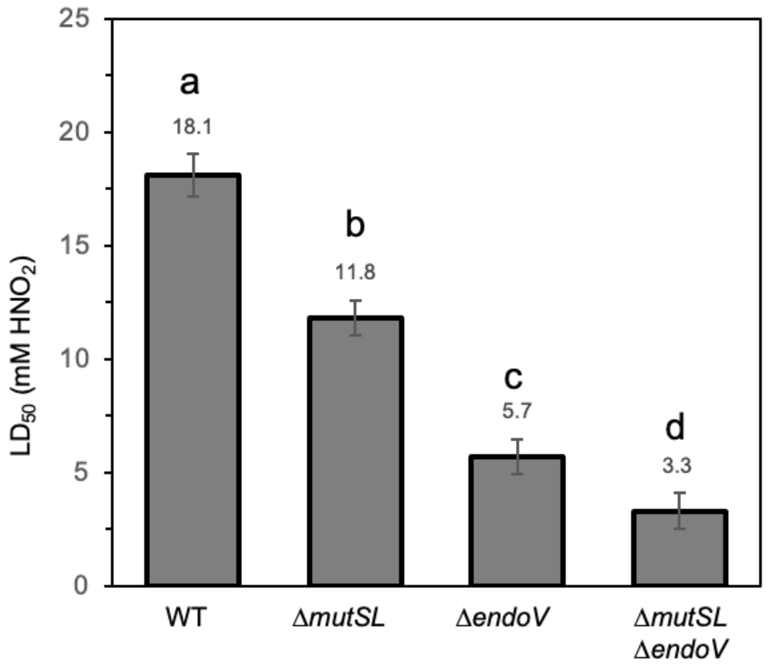

Supplementary Figure 1. **Median Lethal Doses (LD_{50}s) to HNO_2 calculated from dose-response curves of *B. subtilis.*** WT, *mutSL*, *endoV* and *mutSL endoV* strains were grown in A3 medium to a OD_{600nm} of 0.5 and then treated with different doses of nitrous acid (HNO_2). Each bar represents the mean of data collected from three independent experiments per triplicate and error bars represent the standard error of the mean. Letters a, b, c and d, indicate significant differences between strains as determined by one-way analysis of variance (ANOVA) followed by a Tukey's post-hoc test; $P < 0.05$

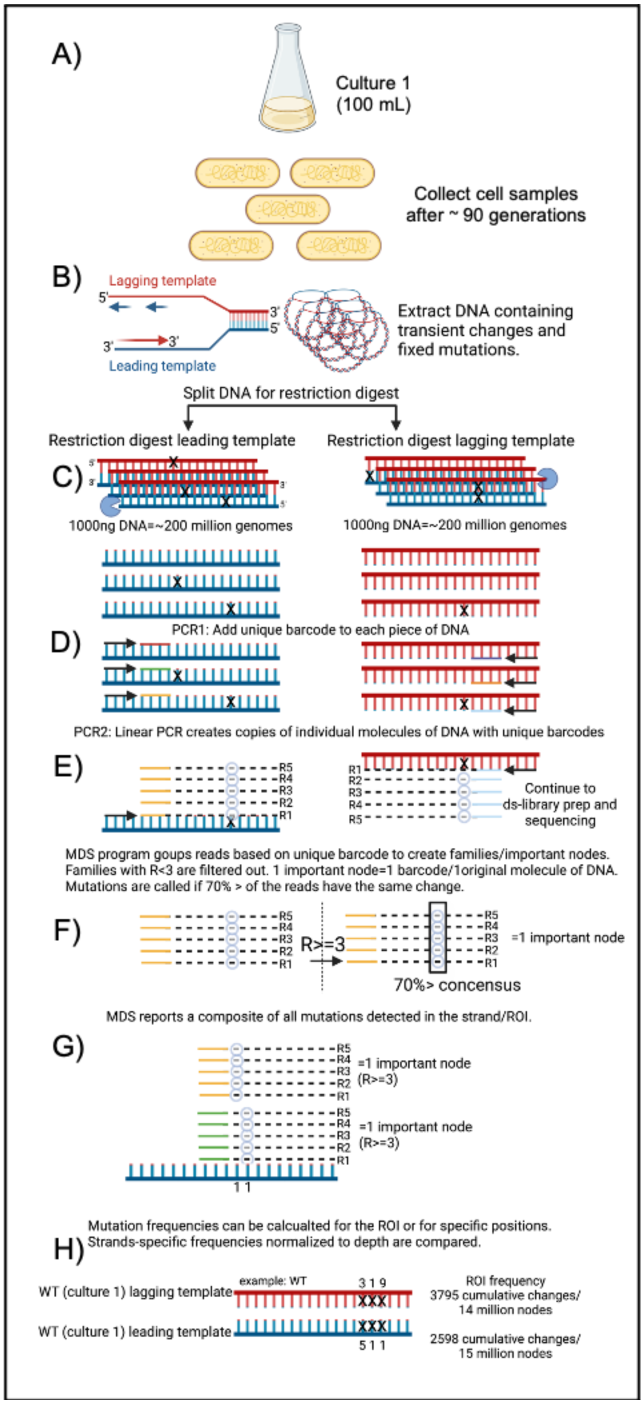

Supplementary Figure 2. **Strategy to generate independent leading and lagging DNA strand libraries. A)** Cultures of *B. subtilis* strains were prepared from isolated colonies and maintained in active growth without selective pressure. A sample of cells was collected after ~90 generations. **B)** DNA in growing cells is captured in active DNA synthesis as shown for the leading (red line) and lagging (blue line) replicating strands; therefore, genomic DNA containing transient changes and fixed mutations is obtained. **C**) Chromosomal DNA is split to prepare independent leading and lagging strand libraries. To this end, the *rpoB* ROI in the leading (blue solid line) and lagging (red solid line) template libraries is delimited by

restriction digestion of 2000 ng (~ 200 million genomes) of DNA with *Nla*III and *Hpy*166III, respectively. **D**) A first round of PCR allows the introduction a unique barcode to each piece of DNA. **E)** A second PCR create copies of the individual molecules of DNA with unique barcodes, obtaining double-strand DNA. The libraries are amplified by 14 rounds of PCR with appropriate adapters and finally are subject to sequencing. **F)** To analyze the data, MDS program groups reads based on unique barcode to create families or important nodes. This means 1 important node= 1 barcode/ 1 original molecule of DNA. Family with R≤3 is filtered out. Mutations are called if ≥70% of reads have the same change in the same family. **G)**. The MDS report is composed by all mutations detected in the strand/ROI. This means, each family or important node contribute with the changes of one specific position. The changes in the same position of different families are cumulative. **H)** Mutation frequencies can be calculated for the full ROI or for specific positions. Strands-specific frequencies normalized to their depth are compared. All libraries have different depth to be normalized, *vgr*, to calculate the WT lagging template strand mutation frequency, we consider 3795 cumulative changes in all ROI normalized with a depth of 14 million nodes, while to calculate the leading template strand, we consider 2598 cumulative changes with a depth of 15 million nodes.

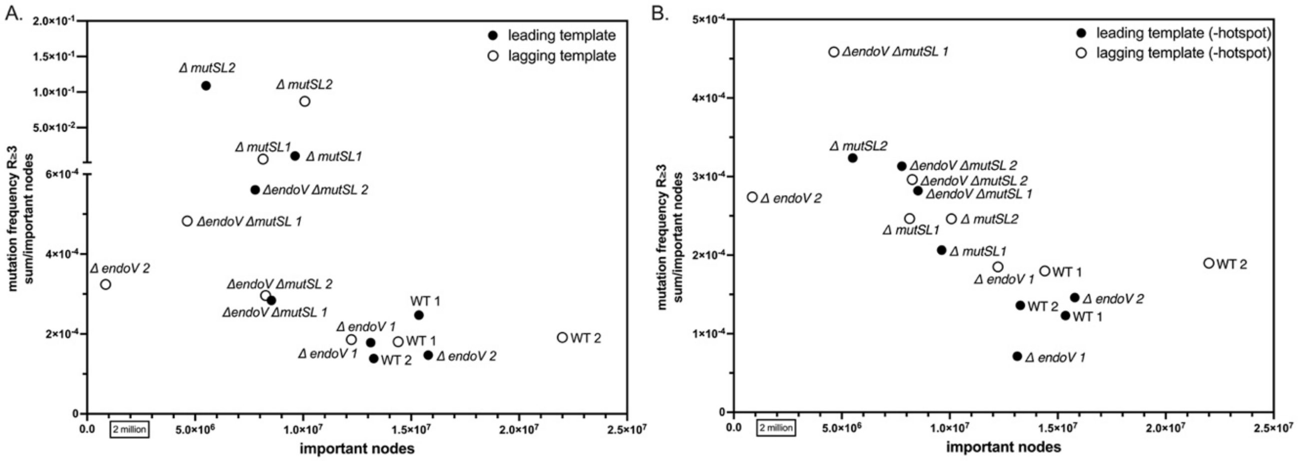

Supplementary Figure 3. **Summary of MDS libraries.** Plots show the mutation frequency (sum of all the mutations/important nodes) of every MDS library sequenced. Mutation frequency is the sum of all the mutations in the 68-bp ROI, included **(A)** or excluded **(B)** the hotspot (14), divided by important nodes.

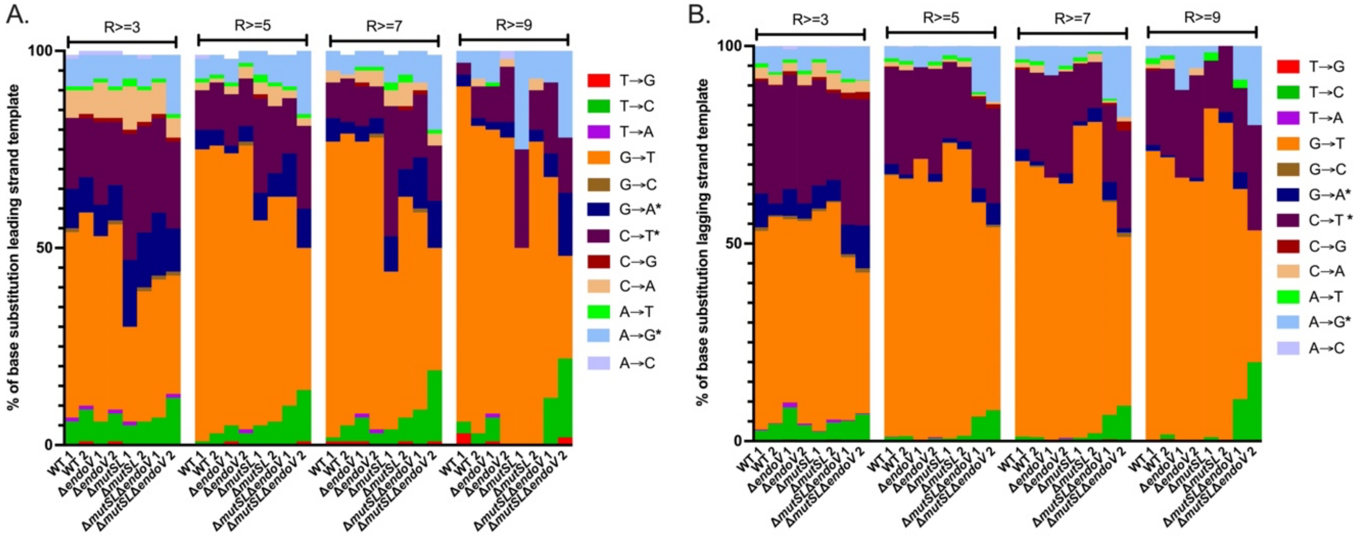

Supplementary Figure 4. MDS analysis of the base substitution spectrum occurring in the leading (A) and lagging strands (B) templates of the *rpoB* ROI at different R values. Percent of the profile of base mutations occurring in the *rpoB* ROI determined by MDS as a function of the R values. MDS was applied to genomic libraries from two independent cultures that were obtained from strains *B. subtilis* WT, Δ*endoV*, Δ*mutSL*, and Δ*endoV* Δ*mutSL,* the number after strain´s genotype indicate the replicate. *, indicate putative base-deamination promoted base substitutions.

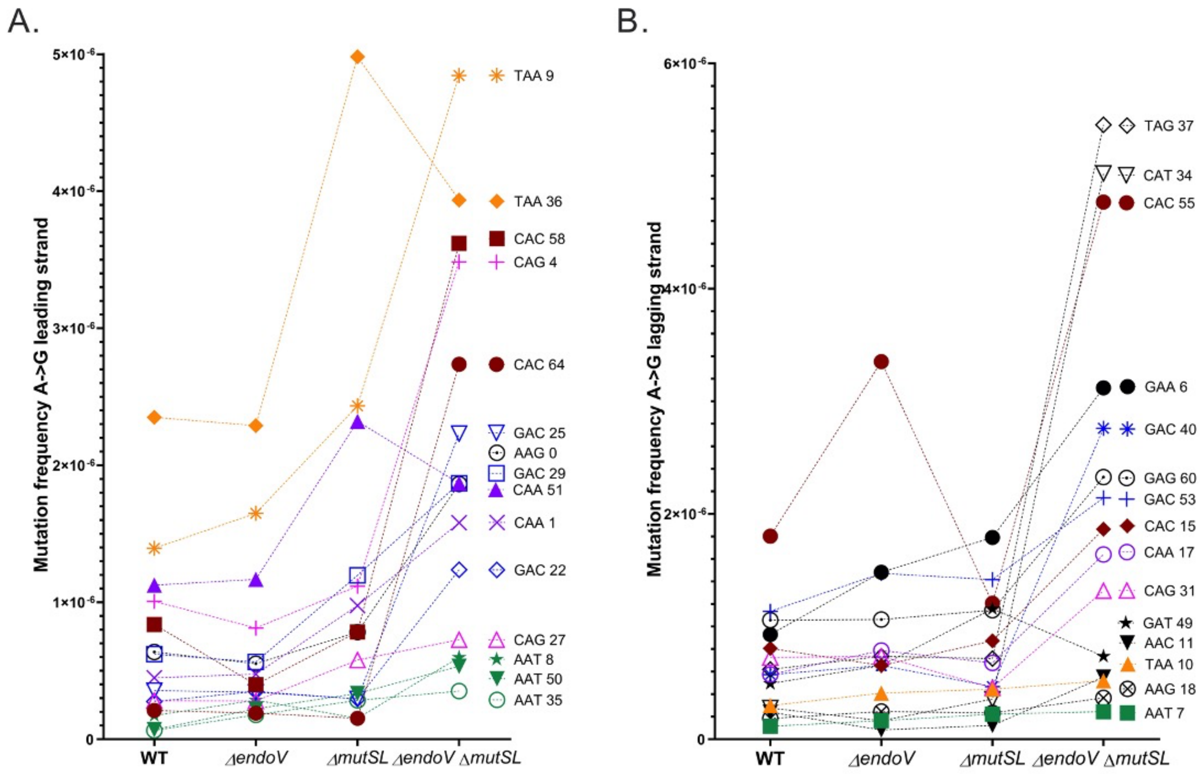

Supplementary Figure 5. **Effect of the sequence context (specified as deoxy trinucleotides) on the mutation frequency associated to A→G substitutions**. Leading (**A**) and lagging (**B**) strands of the 68-bp ROI in the *B. subtilis* strains are indicated. The number next to each triplet indicate the position of the mutated A in each DNA strand.

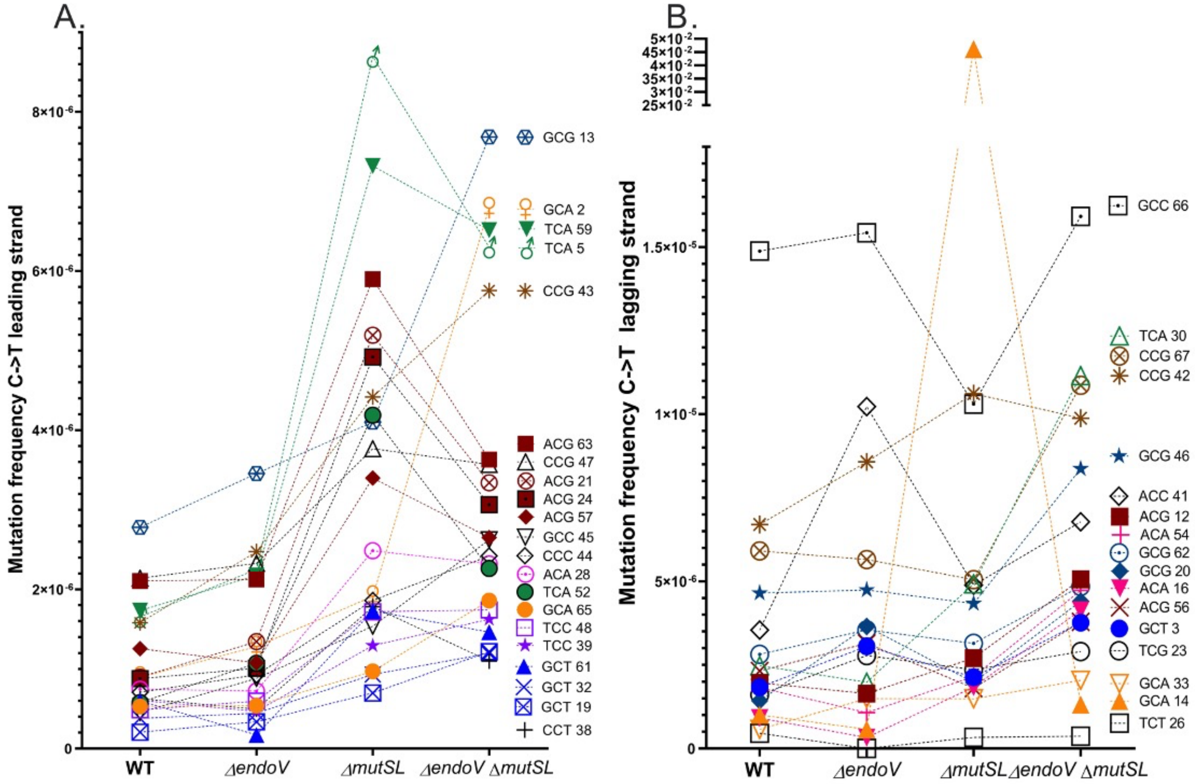

Supplementary Figure 6 Effect of the sequence context (specified as deoxy trinucleotides) on the mutation frequency associated to C→T substitutions. Leading (**A**) and lagging (**B**) strands of the 68-bp ROI in the *B. subtilis* strains indicated. The number next to each triplet indicate the position of the mutated C in each DNA strand.

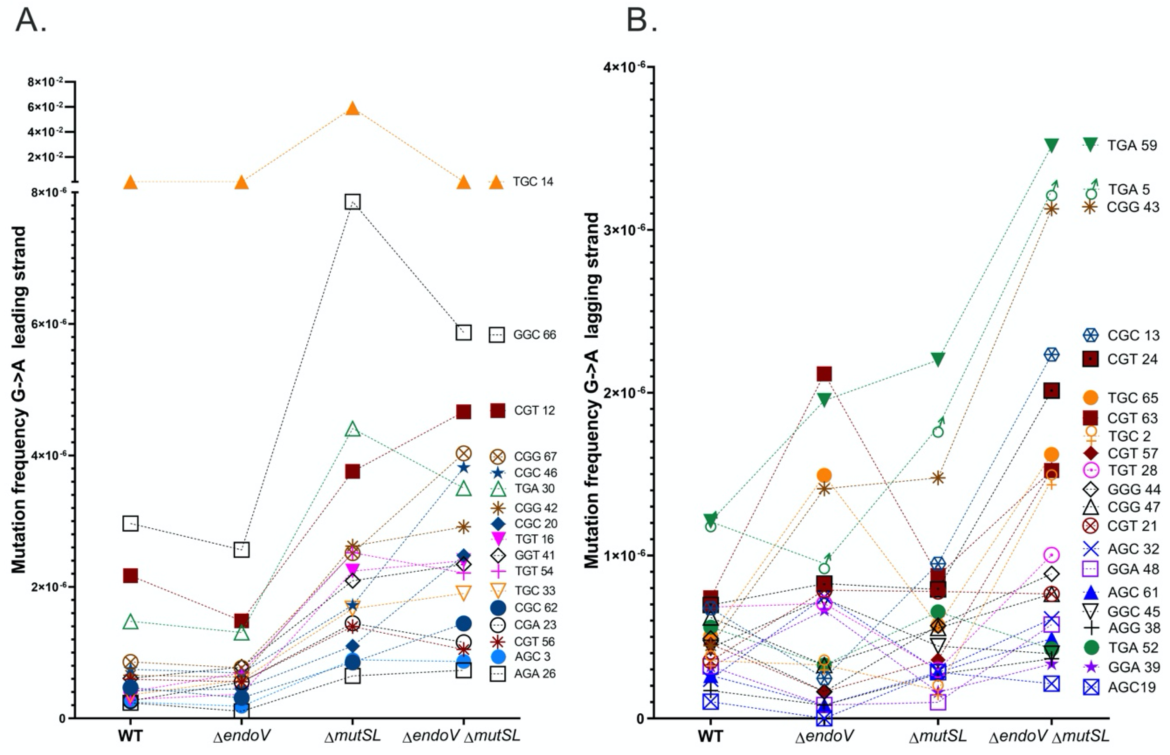

Supplementary Figure 7. Effect of the sequence context (specified as deoxy trinucleotides) on the mutation frequency associated to G→A substitutions. Leading (**A**) and lagging (**B**) strands of the 68-bp ROI in the *B. subtilis* strains are indicated. The number next to each triplet indicate the position of the mutated G in each DNA strand.

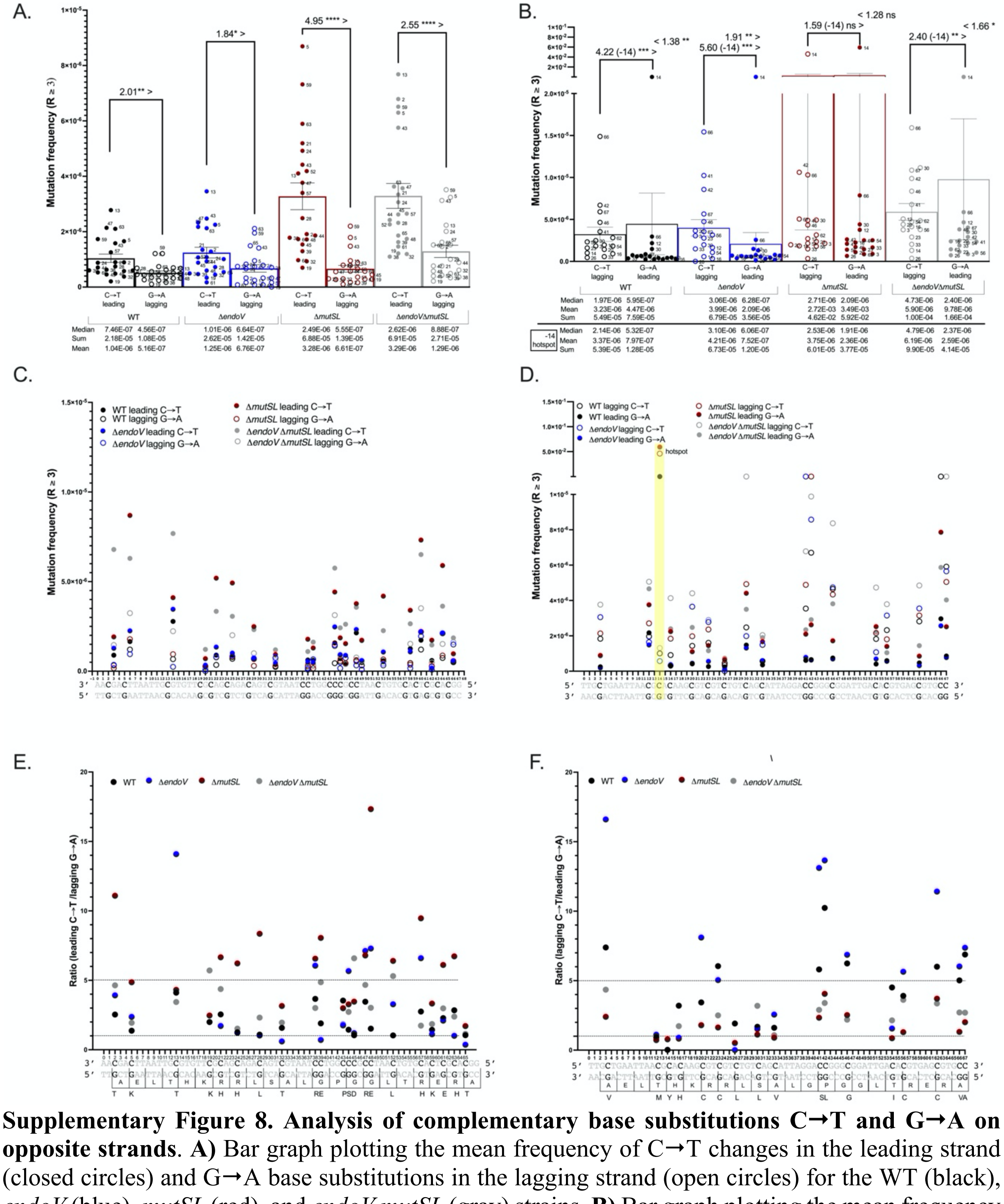

Supplementary Figure 8. Analysis of complementary base substitutions C→T and G→A on opposite strands. **A)** Bar graph plotting the mean frequency of C→T changes in the leading strand (closed circles) and G→A base substitutions in the lagging strand (open circles) for the WT (black), *endoV* (blue), *mutSL* (red), and *endoV mutSL* (gray) strains. **B)** Bar graph plotting the mean frequency of C→T changes in the lagging strand (open circles) and G→A base substitutions in the leading strand (closed circles) for the WT (black), *endoV* (blue), *mutSL* (red), and *endoV mutSL* (gray) strains. For A and B, the median, mean, and sum are calculated considering all positions in the ROI (dots) and every dot represents the mean of two replicates. The bars plot the mean and the error bars are SEM. To calculate the fold difference between the strands, the C→T frequency mean was divided by the

G→A frequency mean or vice versa depending on which value was greater than 1. Significance was determined using a Mann-Whitney test that considered all the positions in the ROI. **C**) Plots the C→T leading strand frequency and its complementary G→A lagging strand frequency of each strain across the ROI. The lagging strand is shown in open circles while the leading strand is shown in closed circles. **D**. Plots the C→T lagging strand frequency and its complementary G→A leading strand frequency for each strain across the ROI. Every dot represents the mean of two replicates. **E-F**) Analysis of fixed/transient C→T, G→A mutations in the lagging (above) and leading (below) strand libraries of the *rpoB* ROI determined by the C→T/G→A ratio. As the ratio approaches a value of 1.0, there is a higher probablity that the C-T transition became fixed in the corresponding strand; in contrast C→T/G→A ratios with values >1.0 are considered transient mutations. Ratio values represent the average frequency from two independent biological replicates at each strand and position. Letters in the bottom of X-axis indicate predicted amino acid changes that may result if the indicated mutation was fixed.

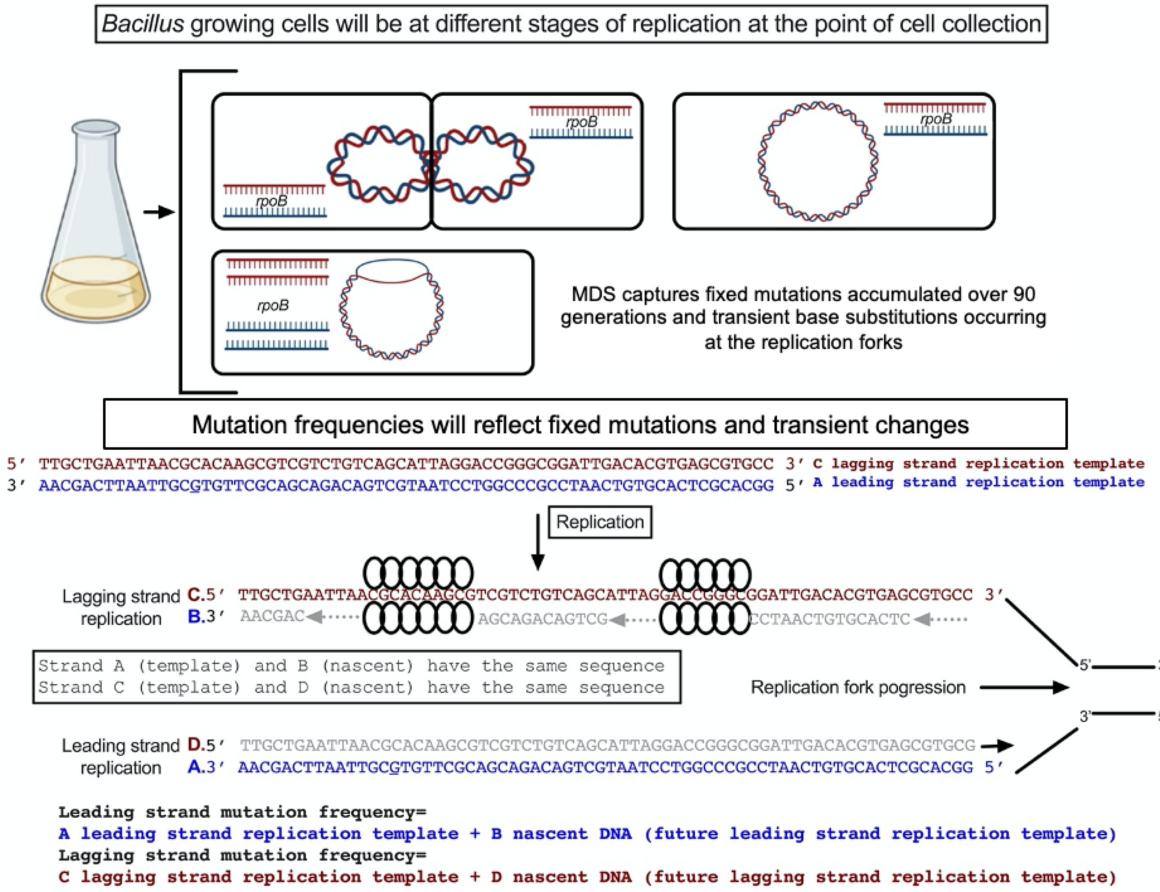

Supplementary Figure 9. **Schematic explaining MDS data interpretation for the leading and lagging strand mutation frequencies.** Above, actively growing cells from a *B. subtilis* culture allows MDS to capture fixed mutations accumulated for ~90 generations and transient base substitutions occurring at the replication fork. Below, Primers are designed to capture the leading strand mutation frequencies composed by (A) leading strand replication template and (B) nascent DNA (future leading strand replication template). Likewise, primers designed to capture the lagging strand mutation frequency composed by (C) lagging strand replication template and (D) nascent DNA (future lagging strand replication template) since they have the same sequence. We consider this schematic when interpreting the results from MDS.

Supplementary Table 1. Bacterial strains and plasmid used in this study.

Strain or plasmid	Genotype and description	Source or reference
**Strains**		
B. subtilis		
168	Wild Type, *trpE*	Laboratory stock
PERM647	Δ*mutSL::neo;* Δ*endoV::lacZ* Neo^r Em^r	(López-Olmos et al, 2012)
PERM739	Δ*mutSL::neo;* Neo^r	(López-Olmos et al. 2012)
PERM791	Δ*endoV::lacZ;* Em^r	(López-Olmos et al. 2012)

Supplementary References

López-Olmos, K., Hernández, M. P., Contreras-Garduño, J. A., Robleto, E. A., Setlow, P., Yasbin, R. E., et al. (2012). Roles of endonuclease V, uracil-DNA glycosylase, and mismatch repair in *Bacillus subtilis* DNA base-deamination-induced mutagenesis. *J. Bacteriol.* 194, 243–252. doi: 10.1128/JB.06082-11
